# Enhanced emission directivity from asymmetrically strained colloidal quantum dots

**DOI:** 10.1126/sciadv.abl8219

**Published:** 2022-02-23

**Authors:** Yang Song, Ruixiang Liu, Zhibo Wang, Huaiyu Xu, Yong Ma, Fengjia Fan, Oleksandr Voznyy, Jiangfeng Du

**Affiliations:** 1 Hefei National Laboratory for Physical Sciences at the Microscale and School of Physical Sciences, University of Science and Technology of China, Hefei 230026, China.; 2 CAS Key Laboratory of Microscale Magnetic Resonance, University of Science and Technology of China, Hefei 230026, China.; 3 CAS Center for Excellence in Quantum Information and Quantum Physics, University of Science and Technology of China, Hefei 230026, China.; 4 Department of Physical and Environmental Sciences, University of Toronto Scarborough, Toronto, Ontario M1C 1A4, Canada.; 5 College of Photoelectronic Engineering, Chongqing University of Posts and Telecommunications, Chongqing 400065, China.; 6 State Key Laboratory of Advanced Design and Manufacturing for Vehicle Body, Hunan University, Changsha 410082, China.

## Abstract

Current state-of-the-art quantum dot light-emitting diodes have reached close to unity internal quantum efficiency. Further improvement in external quantum efficiency requires more efficient photon out-coupling. Improving the directivity of the photon emission is considered to be the most feasible approach. Here, we report improved emission directivity from colloidal quantum dot films. By growing an asymmetric compressive shell, we are able to lift their band-edge state degeneracy, which leads to an overwhelming population of exciton with in-plane dipole moment, as desired for high-efficiency photon out-coupling. The in-plane dipole proportion determined by back-focal plane imaging method is 88%, remarkably higher than 70% obtained from conventional hydrostatically strained colloidal quantum dots. Enhanced emission directivity obtained here opens a path to increasing the external quantum efficiencies notably.

## Teaser

Asymmetric strain lifts the dipole degeneracy and enables directional photon emission in colloidal quantum dots.

## INTRODUCTION

Colloidal quantum dots (CQDs) are appealing active emitting layer candidate for light-emitting diodes (LEDs) thanks to their narrow emission linewidth and wide wavelength tenability (*1*–*3*). The past decades have witnessed tremendous progress in maximizing the energy efficiency of these devices, primarily through optimizing charge injection (*4*–*8*) and improving radiative recombination efficiency (*9*–*11*). The former was achieved through band alignment and mobility engineering in different functional layers of LED, and the latter was enabled by advanced core-shell CQD syntheses and ligand passivation (*11*, *12*). These successful strategies have led to nearly 100% internal quantum efficiency; however, the peak external quantum efficiency (EQE) of well-optimized devices is still limited to 20 to 30% (*5*, *13*) since most of the emitted photons are trapped inside the LED due to the low light extraction efficiency (*14*, *15*).

Microcavities, microlenses/arrays, and surface roughening have been used to improve light extraction (*16*–*20*); however, viewing angle becomes compromised, and the fabrication process is significantly more complicated in these approaches (*15*, *21*, *22*). In contrast, orienting the emission dipole moment of the CQDs to converge the photon emission angle is a more feasible strategy to extract light. It neither compromises the viewing angle nor requires additional device processing (*23*).

In previous literature, dipole moment orientation has been demonstrated in nanorods (*24*), dot-in-nanorod (*25*), nanoplates (*26*, *27*), and dot-in-plate nanocrystals (*28*, *29*) with large aspect ratios. Unfortunately, the efficiencies of LED devices made from large–aspect ratio nanocrystals are typically far below the values achieved in LEDs using more isotropic colloidal CQDs (*30*, *31*), which is likely due to either reduced electron and hole wave function overlap, imbalanced charge injection, or exciton quenching induced by ultrafast nonradiative energy transfer. Previous researches also revealed that anisotropic strain is another efficient tool to modulate the electronic structures and the optical transition of the CQDs, and it has been reported that the photoluminescence (PL) lifetime (*32*) and linewidth (*33*), polarization of absorption (*28*), and lasing thresholds (*34*) can be greatly affected.

Here, we report that a pronounced dipole orientation can be achieved in dedicatedly strained CdSe-CdS core-shell CQDs that have similar aspect ratio to CQDs that performed well in LED devices. The cores of the CQDs are strained perpendicular to the *c* axis, namely, biaxially strained, resulting in the lifting of the polarization degeneracy of band-edge states, enabling directional emission from individual CQDs. In addition, thanks to the flat exposed facet, we can prepare solid thin films with CQDs highly oriented by spin-coating. This further enables us to achieve directional light emission from CQD films, paving the way for the fabrication of ultrahigh-efficiency QD LED (QLED).

## RESULTS

### CdSe QD fine structure and polarization

Excitons with dipoles parallel to the CQD film plane, namely, in-plane dipoles, are desired for light out-coupling because they emit photons perpendicular to the film substrate, facilitating their escape into air. Those with dipoles perpendicular to the film plane (so called out-of-plane dipoles) emit photon parallel to the film plane, making light extraction difficult (*14*). Our transfer matrix calculation (*35*, *36*) reveals that the proportion of in-plane dipole plays a role as important as PL quantum yield (PL QY) in determining the EQE of QLED (fig. S1).

Thus, we sought to find CQDs that would generate excitons with pronounced in-plane dipoles. Effective mass, k*p models (*37*), and semiempirical pseudopotential (*38*) or tight-binding methods (*34*, *39*) provide a comprehensive understanding of the band structure of bulk II-VI and III-V materials, highlighting that light holes and heavy holes are typically built from atomic p-orbitals of different orientation. Sufficiently splitting these levels by quantum confinement (*40*) or by asymmetric strain (*28*), so that only one of these levels remains thermally populated, may allow for directional light emission. However, the direction of strain is critical to push the transition with desired dipole orientation lower in energy.

**Fig. 1.**
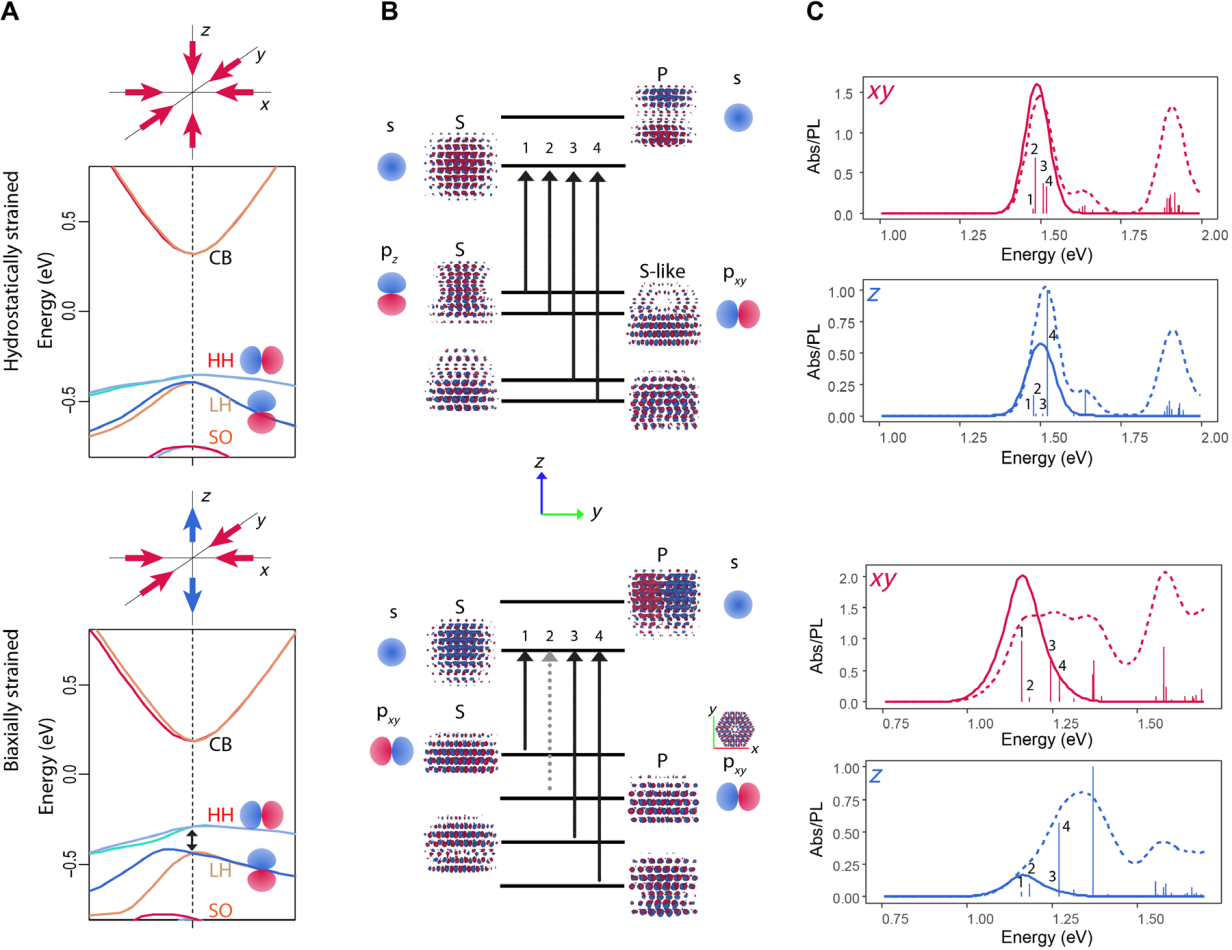
DFT-calculated band structures and optical transitions of CdSe CQDs. (**A**) Bulk band structure of hydrostatically and biaxially strained wurtzite CdSe, (**B**) wave functions of several band-edge states in a 3-nm CdSe CQD, and (**C**) absorption and PL for *XY* polarization (red) and *Z* polarization (blue) of a hydrostatically strained and biaxially strained 3-nm CdSe CQD. CB, conduction band; HH, heavy hole; LH, light hole; SO, spin-orbit coupling; Abs, absorption; PL, photoluminescence.

To find the optimal strain direction and required strain strength, we performed density functional theory (DFT) computations including spin-orbit coupling for 3-nm wurtzite CdSe nanoparticles, hydrostatically and biaxially strained (Fig. 1). Calculated transition dipole moments show that in hydrostatically strained CdSe CQDs, unlike in CdSe nanoplatelets (*27*), heavy holes and light holes are not sufficiently split. In contrast, we find that a biaxial compression perpendicular to the *c* axis allows achieving sufficient state splitting and *XY* dipole orientation for the emitting state (i.e., CQD emits photon along its *Z* direction, allowing for efficient out-coupling), because the strain effect along this direction is superimposed with the effect of crystal field. Our previous work demonstrated that this kind of strain can be achieved in drum-like wurtzite CdSe/CdS nanocrystals (*34*).

To elucidate the origin of such dipole orientation, we deconstructed the nanoparticle wave functions in terms of their bulk Bloch components and quantum-confined envelopes. The former ones are responsible for transition dipole orientation, whereas the latter affects only the selection rules. The underlying bulk Bloch states in the conduction band are composed primarily of Cd 5s atomic orbitals. Valence band is composed of Se 4p orbitals with a slight admixture of Cd 4d and Cd 5s. Heavy holes and light holes have different orbital orientation (i.e., p*_xy_* versus p*_z_*) but remain nearly degenerate in the hydrostatic strain case (Fig. 1).

Both hydrostatic and biaxial compressions change the overlap of the Se and Cd atomic orbitals, resulting in slight energy shifts. In the former case, because of the uniform compression from different directions, energy shifts in p*_xy_* and p*_z_* orbitals are so close that degeneracy is not lifted. Although in the latter case, because of anisotropic strain, the overlap of the Se p*_xy_* and p*_z_* orbitals with Cd 4d orbitals is affected to a different extent, leading to difference in coupling and, consequently, lifting of the degeneracy. For this particular orientation of the biaxial strain, p*_xy_* orbitals become the highest valence band (Fig. 1A).

With quantum confinement taken into consideration, the conduction band envelopes (Fig. 1B) have classical particle-in-a-box S and P shapes due to only one electron band with low effective mass contributing to these states. Valence band envelopes are more complicated due to spin-orbit effects and due to the mixing of several proximate bands with different effective masses, resulting in four nearly degenerate hole states. Some of them have a zero-node (S-like) structure, and others have one or two nodes, corresponding to P-like (donut) and D-like shapes, respectively. The underlying Bloch atomic p*_xy_* or p*_z_* orbitals remain clearly discernible upon zooming in.

Assessing whether the optical transition is allowed or forbidden for a given polarization can be done by looking at whether the involved functions are symmetric or asymmetric. For example, in the case of an S-shaped conduction band envelope and an S-shaped valence band envelope, transition dipole along *XY* will be nonzero only if the involved valence band Bloch functions have a p*_xy_* shape.

Transition dipole moments broadened by a Gaussian distribution allow reproducing the absorption spectra, whereas the inclusion of thermal population allows simulating the PL spectra (Fig. 1C). In the hydrostatic strain case, both *XY* and *Z* excitonic states are equally populated, resulting in a nonpolarized emission. Under compressive biaxial strain perpendicular to the *c* axis, transition with *XY* dipole becomes the lowest in energy, while the transition with *Z* dipole is pushed so much higher that it remains unpopulated. As a result, emission with *XY* dipole becomes nearly 10-fold stronger.

### The oriented assembled CQD films and their polarized absorption

**Fig. 2.**
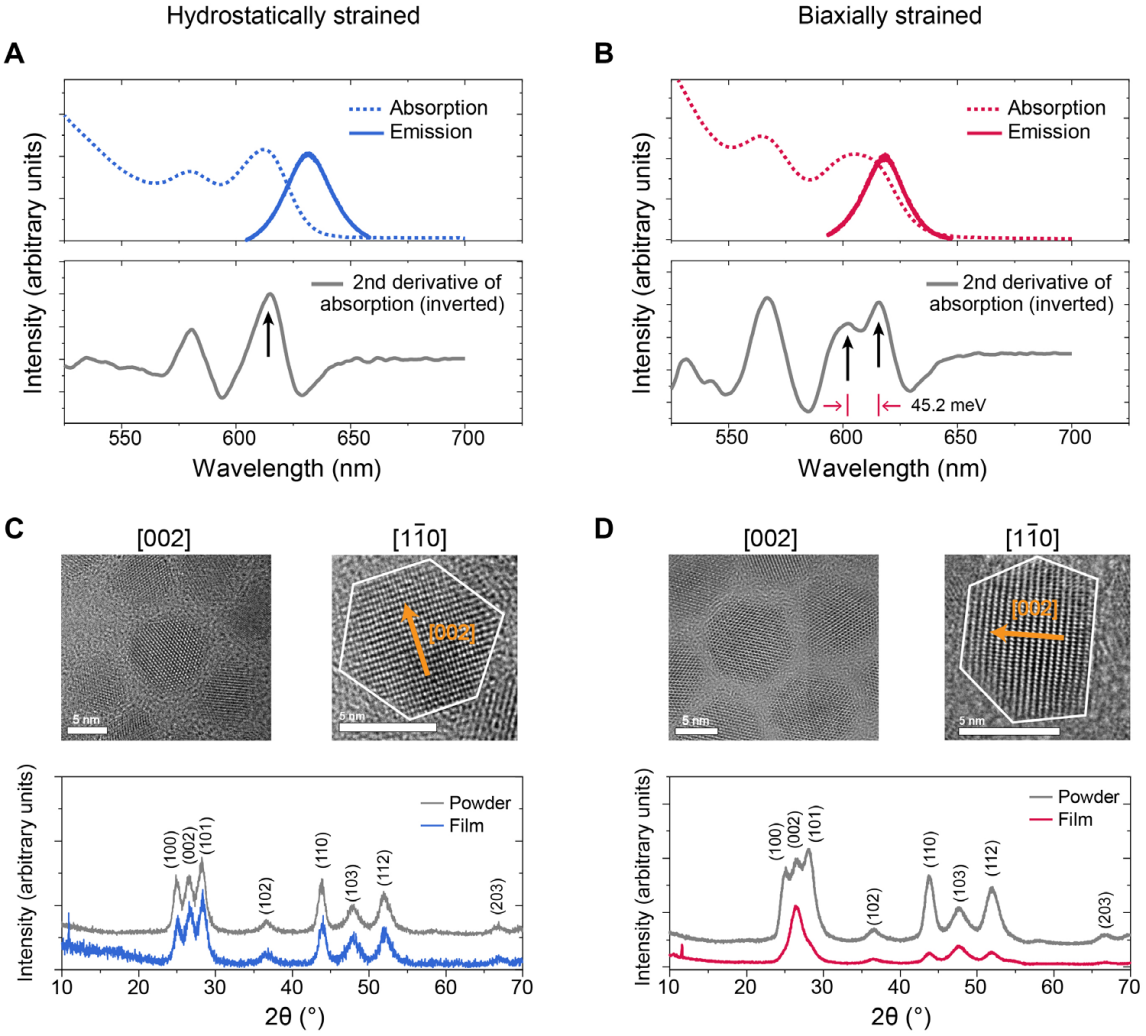
The optical, morphology, and crystalline structure characterizations. The absorbance and PL spectra of the hydrostatically (**A**) and biaxially (**B**) strained CQDs. The second derivative of the absorbance reveals that the broadening of the first exciton peak in biaxially strained CQDs is due to the separation of initially closely spaced peaks, which is not observed in hydrostatically strained CQDs. The crystalline structure and shape of (**C**) hydrostatically and (**D**) biaxially strained CQDs, which are extracted from HRTEM of CQDs on thin carbon film. They have similar size and shape when viewed from the *c* axis ([002] axis), but the differences of shape are observed from the side view ([1$\bar{1}$0] axis). The hat-like shape induces the orientation of biaxially strained CQDs, which is evaluated by comparing the XRD spectra of powder and film samples. The diffraction peak ratio of the (002) to (100) facets is remarkably intensified in biaxially strained CQD film samples, indicating that the *c* axis of most of the biaxially strained CQD is normal to the substrate, but it is not observed in hydrostatically strained CQD films. The XRD spectra of the film sample are obtained from the CQDs drop-casted on nondiffraction silicon substrate.

In light of the above theoretical calculations, we synthesized biaxially strained CdSe-CdS core-shell CQDs by modifying our previous protocol (*34*) in search for directional light-emitting CQDs. The crystalline structures of the core and shell are both wurtzite, and the biaxial strain was introduced by using a mixture of oleylamine and octadecylene as solvent and sulfur dissolved in trioctylphosphine as sulfur precursor during the CdS shell growth process. Since the sulfur-trioctylphosphine has a weaker binding to the (002) facet than the other facets, the shell growth on (002) facet is blocked by oleylamine, while the growth on the other facets keeps going, leaving (002) facet as the flat dominant exposing facet (Fig. 2, C and D). This not only leads to anisotropic compressive strain because of the lack of shell material on (002) facet but also facilitates the oriented assembly of CQD during the film preparation process. We also synthesized hydrostatically strained CQDs by following an existing procedure (*41*) for comparison. The main difference is that a much stronger binding sulfur precursor, i.e., octanethiol, is used to grow more uniform CdS shell to achieve hydrostatic strain.

Biaxial strain–induced perturbation to the band-edge states can be observed from the absorption spectra. The first exciton peak is gradually broadened during the shell growth process (Fig. 2B and fig. S11), while it is not observed in hydrostatically strained CQDs (Fig. 2A). The second derivative of absorbance spectra reveals that the broadening in the first exciton peak is due to the splitting of exciton states, which, according to previous and our own theoretical simulations, are related to light and heavy holes (*37*, *42*), and a similar phenomenon has been observed in dot-in-plate nanocrystals (*19*). The splitting reaches 45.2 meV in our final CQD, which is much bigger than the thermal activation energy (~26 meV) at room temperature, and therefore, the thermal population of higher energy state can be suppressed. The size of biaxially strained CQD along [002] and its perpendicular zone axis are close (figs. S12 and S18), indicating that the total shell thickness along these two directions is also similar; therefore, to produce sufficient asymmetric biaxial compression, core QDs should be off-centered within the shell, as we reported previously (*34*); otherwise, the core QDs should be close to hydrostatically strained. The lifting of degeneracy played an important role in lowering the lasing threshold (*34*, *43*), narrowing the PL, and modulating the polarization of absorption (*28*) in previous reports; in this work, we are particularly interested in its effect on dipole moment orientation.

According to our DFT simulation, the exciton transition dipole moment of the ground state is perpendicular to the *c* axis of the individual CQD, allowing directional light emission along the *c* axis. To retain it in CQD thin films, it is imperative to uniformly orient the *c* axis of CQD to be perpendicular to the substrate. By adopting a simple spin-coating method, we can obtain the preferable orientation of the nanocrystals on glass substrates. A comparison of the x-ray diffraction (XRD) patterns of the spin-coated thin film on nondiffraction silicon wafer and ground powder (Fig. 2, C and D) reveals that the diffraction peak attributed to (002) facet has been markedly intensified in thin-film samples, indicating that most CQDs lie on the substrate with their *c* axis perpendicular to the substrate. This oriented assembly can be well retained on top of a thin layer of poly[(9;9-dioctyluorenyl-2,7-diyl)-co-4,4-(*N*-(4-*s*-butylphenyl)diphenyl-amine)] (TFB) (fig. S13), which is one of the mostly used hole transport layers for high-performance QLED (*8*). In contrast, the XRD patterns of thin films made of hydrostatically strained CQDs resemble that of the powder, suggesting that CQDs are not uniformly oriented (Fig. 2C).

To investigate the inner mechanism of oriented assembly, we performed further analysis on high-resolution transmission electron microscope (HRTEM) images of two types of QDs. These two types of QDs represent a different cross-sectional shape while observing from the [1$\bar{1}$0] axis (Fig. 2, C and D). While the (002) facet is the dominantly exposed facet for biaxially strained QDs, the hydrostatically strained QD shows no preference in exposing (002). When we make films, these flat (002) facets can have close contact with the substrate to reduce the interface area and therefore minimize the surface Gibbs energy. This conclusion is supported by our observation from HRTEM images that (002) is the most commonly observed facet from biaxially strained CQDs (figs. S16 and S19). We further observed from the biaxially strained samples that there are few much smaller CQDs that have elongated shape along the [002] axis, since the dominantly exposed facets are the ones perpendicular to the (002) facet, and their [002] axis is parallel to the substrate rather than perpendicular (fig. S16). This further supports our conclusion that the orientation of CQD is mainly determined by the dominantly exposed facets.

**Fig. 3.**
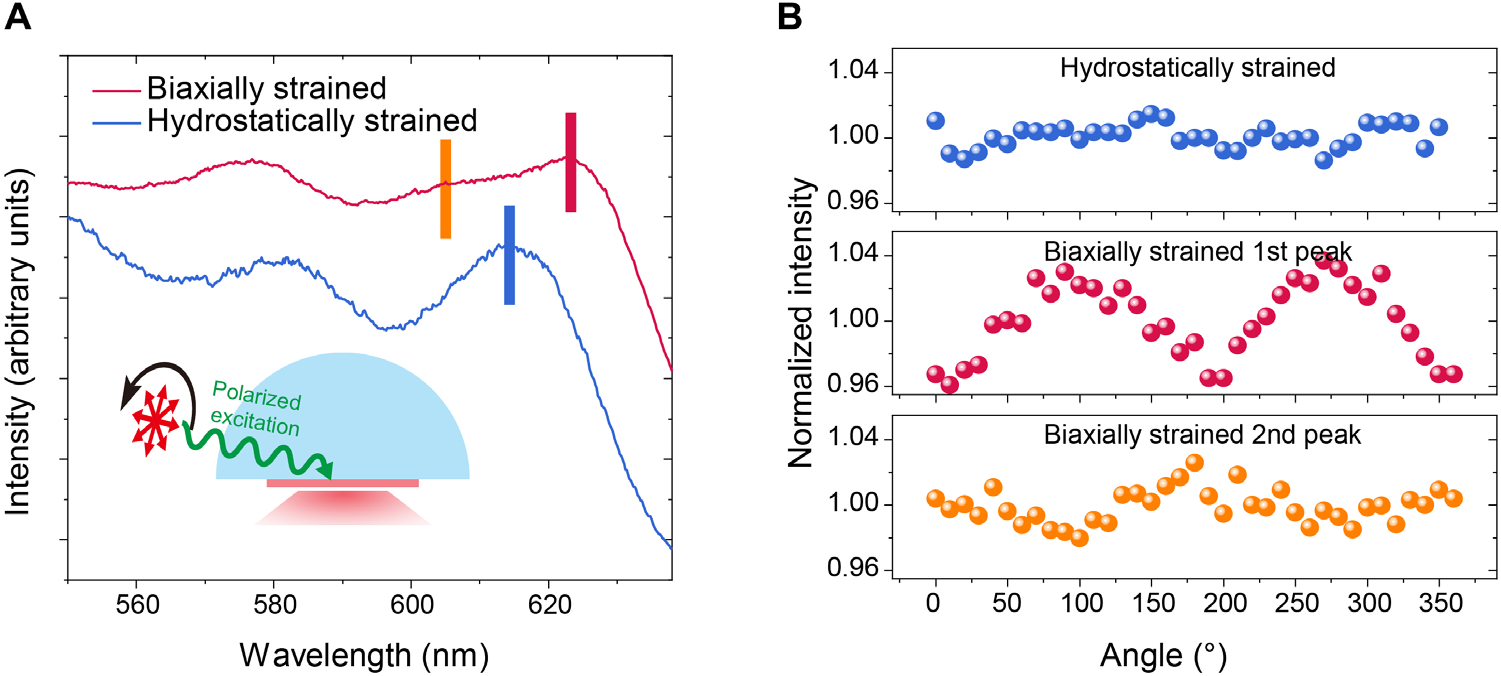
Polarization-dependent PLE measurements. (**A**) The PLE spectra of biaxially strained and hydrostatically strained CQD films. The inset shows the experimental schematic of the polarization-dependent PLE measurements: A semicylindrical lens is connected to a glass substrate by the index-matching fluid to eliminate the impact of substrate surface, and the excitation light is incident from the grazing angle of the substrate. Polarization of excitation light is introduced by placing a polarizer at the outlet of the monochromator. (**B**) The dependence of emission intensity on the polarization direction of excitation. The *x* coordinate is the angle between the polarization direction and the normal direction of the films. The first and second exciton peaks of biaxially strained CQD films show in-plane and out-of-plane polarizations, respectively, while there is no noticeable polarization in the first exciton peak of hydrostatically strained CQD films.

After confirming the CQD orientation in thin films, we sought to test the transition dipole moment orientation of band-edge excitons by measuring the absorption/emission polarization direction. Since the band-edge absorbance of thin-film samples is low, a polarization-dependent PL excitation (PLE) measurement was adopted (details in Materials and Methods), of which the experimental principle is shown in Fig. 3A. Excitation light was incident at a grazing angle through a semicylindrical lens to avoid unwanted change in its polarization, and index-matching oil was applied between the lens and the substrate to ensure air-free adhesion. A small grazing angle (~10° here) allows us to excite the CQD film with primarily in-plane and out-of-plane polarization, respectively. We observed a strong in-plane polarized absorption from the first exciton peak of biaxially strained CQD films (Fig. 3B), suggesting that emission is potentially intensified in the direction normal to the substrate, a feature that is desirable for boosting the light out-coupling efficiency, and we also observed out-of-plane polarized absorption at the second exciton peak, which agrees well with the aforementioned simulation. In contrast, no noticeable absorption polarization is observed at the first exciton peak of hydrostatically strained CQD films, suggesting the superposition of in-plane and out-of-plane dipoles.

### Quantifying the directional emission of CQD film

**Fig. 4.**
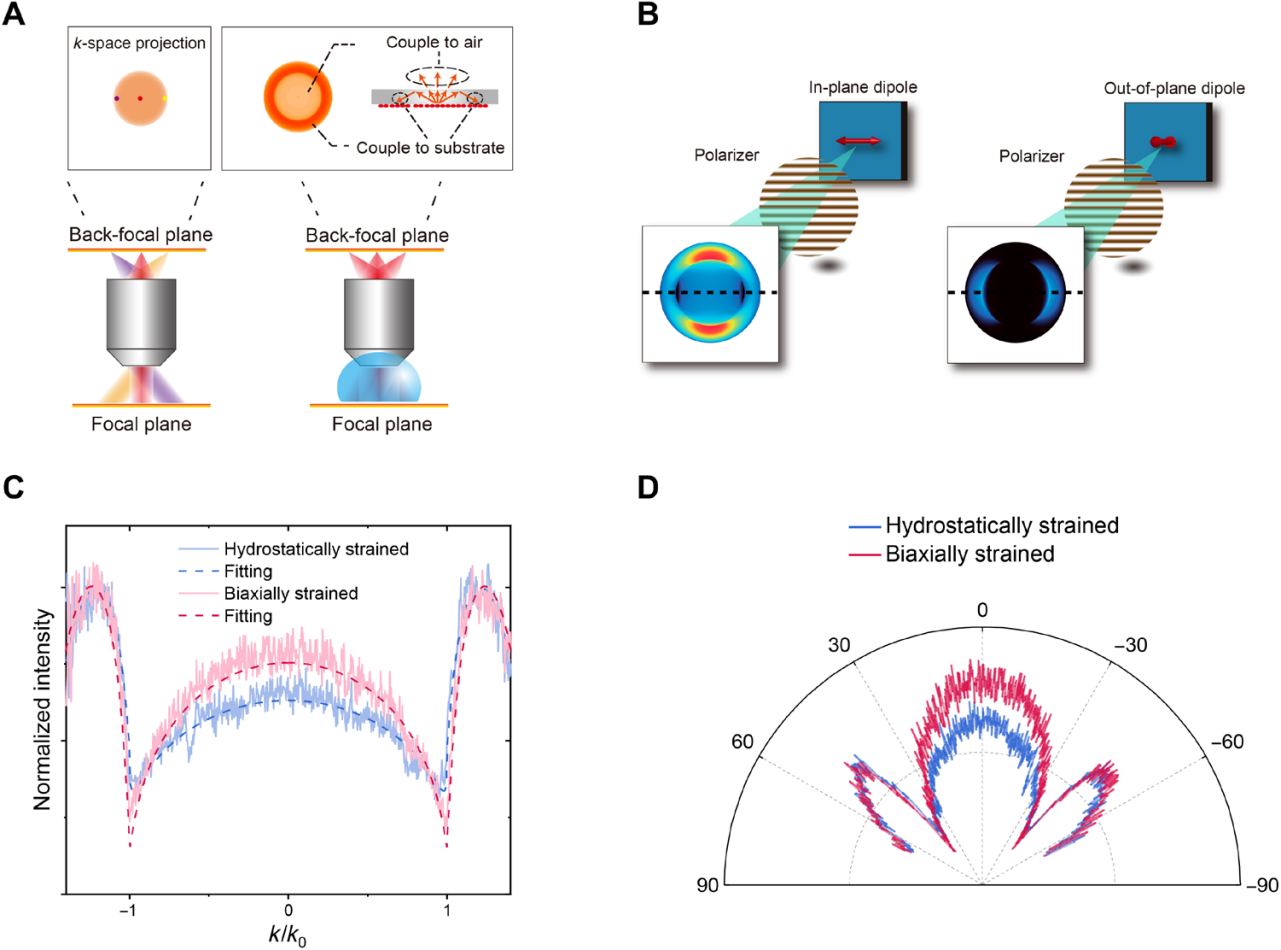
BFP imaging measurements. (**A**) The principle of BFP imaging measurement. Each spot on the BFP corresponds to light with a specific direction incident from the focal plane, and with the help of index-matching oil, we can extend the detection range to include the substrate mode. (**B**) Simulated BFP images of in-plane and out-of-plane dipoles after introducing the polarization. These two images are used as the bases to fit the experimental images to get the proportion of in-plane dipoles in the thin-film samples. (**C**) The intensity profile of experimental BFP images (fig. S4) along the dotted lines indicated in (B). The proportion of in-plane dipole has been remarkably improved from 70% in hydrostatically strained CQD film to 88% in biaxially strained CQD film. (**D**) Intensity profile converted to angular coordinate form. The sample is photoexcited by a 405-nm continuous-wave laser, and then the excitation light is filtered by a band-pass filter.

To further quantify the polarization and the directivity of emission, CQD films are further characterized by a back-focal plane (BFP) imaging method (*26*, *44*, *45*), of which the schematic is illustrated in Fig. 4A. There are real-image planes ahead and behind the objective lens, which are called focal plane and BFP, respectively. With the aid of the lens, light with different propagation directions from the focal plane will be mapped onto different points on the BFP, called a Fourier transformation. The index-matching oil is needed here to collect light with incident angles greater than the critical angle of total internal reflection (Fig. 4A), which is crucial for calculating the proportion of in-plane dipole.

We then need to extract emission direction information, which is determined by dipole orientation, from the BFP images. Filtered by a polarizer, the BFP images are distinctly different for in-plane and out-of-plane dipoles (Fig. 4B). Provided that the refractive index and thickness of the emitting layer and substrate are known, we can calculate the theoretical BFP patterns of two types of dipole and use them as two bases to fit the measured BFP images, and then we can calculate the proportion of the in-plane dipoles, which determines the directivity of the emitter. We first compared BFP images of the submonolayer films of biaxially strained and hydrostatically strained CQDs (Fig. 4C) and found that the proportion of light coupled to the air is considerably enhanced in biaxially strained CQD films. Then, we fit the BFP images and found that the biaxially strained CQD film has a 88% in-plane dipole proportion, contrasting to the value of 70% (67% is isotropic) obtained from hydrostatically strained CQD films. Accordingly, the directivity [defined as *D* = *P*_max_/*P*_mean_ (*46*)] is enhanced from 1.27 to 1.4. We also spin-coated CQDs on glass with a TFB top layer and observed that the enhanced light extraction from biaxially strained CQDs is retained (fig. S13). To further test whether the light extraction can be retained in electroluminescence, we have conducted an angle-resolved electroluminescence measurement in LED with an architecture of sapphire/ITO/PEDOT:PSS/TFB/CQDs/ZnO NPs/Al, and the experimental results prove that enhanced light extraction can be achieved in biaxially strained CQDs (fig. S14). With the improved in-plane dipole proportion and directivity, our transfer matrix calculations show that the out-coupling efficiency of a bottom-emitting QLED (with an architecture of glass-anode-HTL-QD-ETL-cathode) (*13*) could be improved by ~9% (fig. S1).

Note that, besides the electronic structure, the dielectric effect caused by anisotropic shape could also play a role in determining the dipole orientation (*29*). Nevertheless, we do not think that the directional emission of our CQD films is mainly due to the dielectric effect because our CQDs are close to isotropic in dimension. Furthermore, according to Landau’s continuum theory (*47*), with the pure dielectric contrast effect, the in-plane dipole proportion of CQD with a similar aspect ratio as ours can only reach ~68% (text S3). We thus conclude that the dipole orientation in our CQDs is mostly due to the electronic structure of the CQDs arising from the intrinsic biaxial strain.

## DISCUSSION

In this work, we demonstrate that, with the presence of intrinsic biaxial strain that is caused by the off-centering of core CQD inside the compressive shell, near-spherical CdSe/CdS CQDs can serve as directional light emitters. The exposed flat (002) facets allow us to spin-coat films with CQDs uniformly orientated. DFT computations have explicated the origin of biaxial strain–induced directivity. The bond lengths along different crystalline axes have been changed due to biaxial strain, affecting the overlap of atomic orbitals from neighboring atoms and thus the energy of orbitals, lifting the band-edge degeneracy, and setting the in-plane polarized exciton as the ground state. These findings demonstrate a deliberate control over the band-edge states in II-VI CQDs, paving the way for ultrahigh EQE LED devices.

## MATERIALS AND METHODS

### DFT computation

The structures were first optimized using Perdew–Burke–Ernzerhof (*48*) exchange correlation with double-zeta plus polarization basis set using the Siesta code (*49*). To avoid ghost states in computation, the Se pseudopotential was truncated at the s, d, and f orbitals to 1.90, 2.88, and 2.88, respectively. To fully replicate the expected splitting caused by strain, spin-orbit coupling was included in the computation (*50*).

To simulate the QD, a crystal of wurtzite CdSe was shaped to approximately 3 nm. The surface was passivated by pseudo-hydrogens to maintain charge balance. To simulate biaxial strain without an explicit shell, the respective axes were scaled down by the desired factor, the surface atoms were fixed in those positions, and the rest of the atoms were allowed to fully relax. The resulting dot’s wave functions around its bandgap were then investigated for their contributions to optical properties. Optical transition dipole moments were computed by integrating the orbital wave functions saved on the grid using the DenChar utility since Siesta currently does not support the calculation of optical properties for the spin-orbit case natively.

### Chemicals and synthesis

Cadmium oxide (>99.99%), sulfur powder (S, >99.5%), selenium powder (Se, >99.99%), oleylamine (>98% primary amine), octadecene (90%), oleic acid (90%), trioctylphosphine (90%), trioctylphosphine oxide (99%) and octadecylphosphonic acid (97%), and 1-octanethiol (>98.5%) were purchased from Sigma-Aldrich and used without further purification; hexane (anhydrous, 97%) and acetone (99.5%) were purchased from Greagent. The biaxially strained CQDs were synthesized using a modified protocol of our previous work (*34*). A total of 2.9 ml of CdSe CQDs in hexane dispersion was added to a mixture of 12 ml of octadecene and 12 ml of oleylamine in a 250-ml flask. Then, the mixed solution was placed in vacuum at 100°C to evaporate the hexane, followed by heating the solution to 300°C and kept for 0.5 hours to get the CdSe-octadecene-oleylamine dispersion. Diluted Cd-oleate octadecene and trioctylphosphine sulfide octadecene solutions were injected simultaneously and continuously to the as-prepared CdSe solution at a rate of 3 ml/hour (the splitting of band-edge absorption peak during this process is shown in fig. S11). Last, we use the same procedure to purify the core-shell CQDs as in the literature (*34*).

Beyond the asymmetric shell is the thin uniform shell, and it was grown as follows: 1 ml of Cd-oleate diluted in 4 ml of octadecene and 107 μl of 1-octanethiol diluted in 6 ml of octadecene were continuously injected at a speed of 12 ml/hour to grow the second shell. The reaction temperature was elevated to 310°C before injection. After the injection of Cd-oleate into octadecene solution, oleylamine was injected into the solution to improve the dispersibility of the CQDs. Last, the hydrostatically strained CQDs are synthesized by the same method as in the previous work (*34*).

Ensemble absorbance was measured on the MAPADA P4 ultraviolet-visible spectrophotometer over the excitation range of 400 to 700 nm. HRTEM samples were prepared by adding a drop of the solution of CQDs onto an ultrathin carbon film on lacey-carbon support film (D11032) and imaged using a JEOL JEM 2100F HRTEM operating at 200 kV. The x-ray diffraction data refinement is based on the Rietveld method (*51*), and the preferred orientation model used in the software is modified March’s function (March-Dollase model) (*52*). The PL QY is measured using the integrating sphere method (*53*).

### Polarization-dependent PLE spectrum

The PLE measurement was performed on a Hitachi F-7100 fluorescence spectrometer. For measuring the absorption polarization direction of the CQD film (*54*), a Thorlabs WP25M-VIS polarizer was placed at the exit of the excitation light to generate a beam of excitation light with different polarization direction, a semicylindrical quartz lens is used to introduce excitation light from the back of the glass substrate to prevent the glass slide from changing the polarization of excitation light, and a drop of immersion oil (Nikon Type F) was applied between the glass substrate and the semicylindrical lens to get an air-free contact (Fig. 3A). The excitation light is incident at a small angle (~10° relative to the CQD film). By rotating the polarizer, the angle between the polarization of the excitation light and the CQD film plane can be adjusted to get the in-plane to out-of-plane excitation. To eliminate the unwanted excitation polarization anisotropy (*54*), the PLE spectra were normalized by the anisotropic factor (fig. S7).

### BFP imaging method

The CQD submonolayer films were made by spin-coating the CQD in *n*-octane dispersions on a cover slide. An objective lens (Nikon CFI Plan Apochromat Lambda 100× oil, numerical aperture of 1.45) was used to collect real-space and *k*-space emission patterns of the CQD films. To image *k*-space (BFP), two coupled 200-mm focal plano-convex lenses were placed on the back optical path of the objective lens and projected the BFP image of the objective lens onto a sCMOS (scientific complementary metal-oxide semiconductor) camera (TUCSEN Dhyana 400BSI V2). A polarizer (Thorlabs CCM1-PBS251) was used to generate the polarization image of the BFP, and a band-pass filter (Semrock FF02-641/75-25) was used to filter out the excitation and noise light. See text S2 for the BFP image fitting details.

### Optical calculations and BFP fitting

The optical efficiency of LED device is calculated by the transfer matrix method (*35*, *36*), and the BFP fitting is also based on the same method. The influence of the dielectric effect on dipole orientation is calculated according to the continuous medium theory (*47*). All the details of optical calculations and fittings can be found in texts S1 to S3.

## Supplementary Materials

### This PDF file includes:

Supplementary Texts S1 to S3 Table S1 Figs. S1 to S19
