# Supplementary material for: Enhanced emission directivity from asymmetrically strained colloidal quantum dots
Source: Sci Adv. 2022 Feb 23;8(8):eabl8219. doi: 10.1126/sciadv.abl8219 (PMC8865764; doi:10.1126/sciadv.abl8219)
Supplement: Supplementary file 1 — Supplementary Texts S1 to S3 Table S1 Figs. S1 to S19 [file sciadv.abl8219_sm.pdf]

Science Advances
AAAS

Supplementary Materials for

Enhanced emission directivity from asymmetrically strained colloidal quantum dots

Yang Song, Ruixiang Liu, Zhibo Wang, Huaiyu Xu, Yong Ma, Fengjia Fan*, Oleksandr Voznyy*, Jiangfeng Du*

*Corresponding author. Email: ffj@ustc.edu.cn (F.F.); o.voznyy@utoronto.ca (O.V.); djf@ustc.edu.cn (J.D.)

Published 23 February 2022, *Sci. Adv.* **8**, eabl8219 (2022)
DOI: 10.1126/sciadv.abl8219

This PDF file includes:

Supplementary Texts S1 to S3
Table S1
Figs. S1 to S19

Supplementary Text 1

Calculation of the out-coupling efficiency

The transfer matrix method has been elaborated in previous articles (see references of 'Materials and Methods' section), and we recommend readers refer to the original works for details. Here we skip some part of the derivation and only briefly explain the basic principle. For typical multilayer QLED, the glass substrate is several orders of magnitude thicker than the other layers and can therefore be considered infinitely thick, so that the entire structure can be reduced to a few layers sandwiched between two infinitely thick media. As shown in Fig. S1A, the electric field at each virtual plane can be written in the form of a transfer matrix as

$$\begin{pmatrix} E_1^+ \\ 0 \end{pmatrix} = I_1 L_1 \cdots I_{i-1} L_{i-1} \begin{pmatrix} E_i^+ \\ 0 \end{pmatrix} \quad \text{(i<=4)},$$

$$\begin{pmatrix} 0 \\ E_i^- \end{pmatrix} = L_i I_{i+1} \cdots L_6 I_7 \begin{pmatrix} 0 \\ E_7^- \end{pmatrix} \quad \text{(i>=5)}.$$

Here the +/- stands for upward or downward propagation, and $I_i = \begin{bmatrix} 1/t_i & r_i/t_i \\ r_i/t_i & 1/t_i \end{bmatrix}$, $L_i = \begin{bmatrix} \exp(-jk_{zi}d_i) & 0 \\ 0 & \exp(jk_{zi}d_i) \end{bmatrix}$ are the refractive index matrix and the phase matrix, respectively. t_i and r_i are the Fresnel coefficients

$$t_{ij}^p = \frac{2n_i n_j k_{zi}}{n_j^2 k_{xi} + n_i^2 k_{zj}} \ , \ t_{ij}^s = \frac{2k_{zi}}{k_{zi} + k_{zj}} \ ,$$

$$r_{ij}^p = \frac{n_j^2 k_{zi} - n_i^2 k_{zj}}{n_j^2 k_{zi} + n_i^2 k_{zj}} \ , \ r_{ij}^s = \frac{k_{zi} - k_{zj}}{k_{zi} + k_{zj}} \ .$$

E_1^+ and E_7^- is connected by the source term

$$\begin{pmatrix} A_+ \\ A_- \end{pmatrix} = (I_1 L_1 I_2 L_2 I_3 L_3 I_4 L_S)^{-1} \begin{pmatrix} E_{1+} \\ 0 \end{pmatrix} - L_S I_5 L_5 I_6 L_6 I_7 \begin{pmatrix} 0 \\ E_{7-} \end{pmatrix} \ .$$

The source term $\begin{pmatrix} A_+ \\ A_- \end{pmatrix}$ contains the emission dipole's orientation information listed in Table S1 and illustrated in Fig. S2. Then the variation of the power density with E can be expressed in terms of the z-directional component of the Poynting vector

$$S_i = \begin{cases} \text{Real}\left\{ n_i \cos\varphi_i \left(E_{i+} + E_{i-}\right)^* \left(E_{i+} - E_{i-}\right) \right\} & \text{(TE mode)} \\ \text{Real}\left\{ n_i \cos\varphi_i^* \left(E_{i+} + E_{i-}\right)^* \left(E_{i+} - E_{i-}\right) \right\} & \text{(TM mode)} \end{cases} ,$$

where $\cos\varphi_i = \frac{k_{zi}}{k_i} = \sqrt{1 - \frac{k_x^2}{(2\pi n_i/\lambda)^2}}$, $\sin\varphi_i = \frac{k_x}{k_i} = \frac{k_x}{2\pi n_i/\lambda}$. After introducing the dipole orientation,

$$S_i = IP\% \cdot (S_{i,IP,s} + S_{i,IP,p}) + (1 - IP\%) \cdot S_{i,OP,p} \ .$$

In order to reflect the anisotropy of the emission, we convert the Poynting vector to the form of k-coordinate. The power flowing through each interface is the integral of the Poynting vector

$$P_i = \int_0^{\infty} \frac{2\pi}{n_s k_{zs}^2} S_i k_x dk_x \quad .$$

Finally, the out-coupling power is calculated by considering the transmission of P_1 to the air, and the out-coupling efficiency is obtained from the ratio of the power transmitting to air to the total power emitted by the light source: OCE=$P_0/(P_{s'}+P_s)$.

Supplementary Text 2

Fitting method of BFP measurement

The structure used for the fitting is shown in Fig. S3, and the refractive index of each layer is 1.52 (Glass), 1.75 (CQDs), 1 (Air). The glass and air layers are regarded as infinitely thick, and the thickness of CQDs layer is obtained from the fitting of s-polarized profile data. The optical modelling method mentioned in Supplementary Text 1 is also applicable here, the Poynting vector of the virtual plane in glass layer with a specific dipole orientation can be written as

$$S = IP\% \cdot (S_{IP,s} + S_{IP,p}) + (1 - IP\%) \cdot S_{OP,p} \quad , \tag{2-1}$$

so the IP% is obtained by fitting the measured p- and s- direction intensity profile of BFP image using the bases

$$P_p = C \cdot S_p = C \cdot \left[IP\% \cdot S_{IP,p} + (1 - IP\%) \cdot S_{OP,p} \right] \qquad \text{(p-profile)},$$

$$P_s = C \cdot S_s = C \cdot \left[IP\% \cdot S_{IP,s} \right] \qquad \text{(s-profile)}. \tag{2-2}$$

The P and C are respectively the detected emission intensity and the normalized constant. The original BFP images and their cross-section profiles are shown in Fig. S4, and the fitting results obtained by applying equation (2-2) suggest the biaxially strained sample has 88% in-plane dipole while the comparison hydrostatically strained sample has only 70%.

Supplementary Text 3

The dielectric contrast effect of oblate ellipsoid dielectric nanocrystal

We calculated the influence of the dielectric effect on dipole orientation according to the continuous medium theory. The oblate dielectric ellipsoid changes the electric field as expressed by:

$$E_{a,c} = \frac{\epsilon_m}{\epsilon_m + (\epsilon - \epsilon_m)n_{a,c}} E_{0\ a,c}$$

Where E and E_0 is the changed and original electric field, respectively. ϵ and ϵ_m represent the permittivity of the ellipsoid and the surrounding media, a/c is the long- to short-axis ratio of an ellipsoid (as illustrated in Fig. S5A), the value of n can be acquired from the following expression:

$$n_c = \frac{1 + e^2}{e^3}(e - \arctan e)$$

$$n_a = n_b = \frac{1 - n_c}{2}$$

$$e = \sqrt{\left|1 - {a^2}/{c^2}\right|}$$

We calculated the dependence of IP% on the long- to short-axis ratio a/c (Fig. S5B). The results show that with the long/short-axis ratio of about 1.3 in our CQD, in-plane dipole proportion can only reach ~68%. Moreover, even for an ellipsoid with a long/short-axis ratio up to 10, the In-plane dipole proportion is only 76%, far below the value of 88% we got. Therefore, the high emission directionality is mainly due to the biaxial strain induced degeneracy lifting.

Table S1. The source term for different dipole orientation.

	TE-polarized (s)	TM-polarized (p)
In-plane	$\begin{bmatrix} A_+ \\ A_- \end{bmatrix} = \sqrt{\frac{3}{16\pi}} \begin{bmatrix} -1 \\ 1 \end{bmatrix}$	$\begin{bmatrix} A_+ \\ A_- \end{bmatrix} = \sqrt{\frac{3}{16\pi}} \cos\varphi_S \begin{bmatrix} -1 \\ 1 \end{bmatrix}$
Out-of-plane	$\begin{bmatrix} A_+ \\ A_- \end{bmatrix} = \begin{bmatrix} 0 \\ 0 \end{bmatrix}$	$\begin{bmatrix} A_+ \\ A_- \end{bmatrix} = \sqrt{\frac{3}{8\pi}} \sin\varphi_s \begin{bmatrix} 1 \\ 1 \end{bmatrix}$

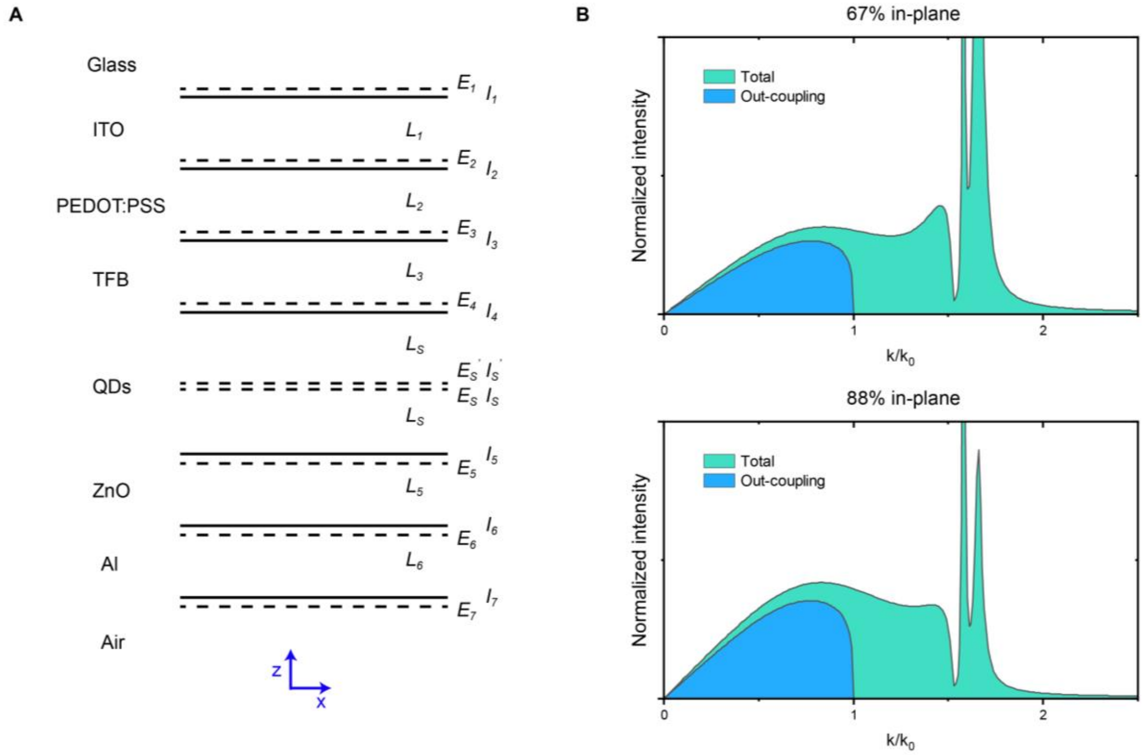

Fig. S1. The optical model for QLED and the power distribution in k-coordinate. (**A**) The LED structure used in the TMM calculation, and the refractive index (at 640nm wavelength) and thickness of each layer is: glass (1.52, infinite thick), ITO (1.87+0.003i, 95nm), PEDOT:PSS (1.5+0.014i, 50nm), TFB (1.68+0.025i, 20nm), QDs (1.75, 40nm), ZnO (1.56+0.01i, 60nm), Al (1.30+7.36i, 100nm), and Air (1, infinite thick). (**B**) The power distribution in k coordinate. By improving the in-plane dipole proportion from 67% (isotropic) to 88%, the out-coupling efficiency can be improved from 30.06% to 39.01%.

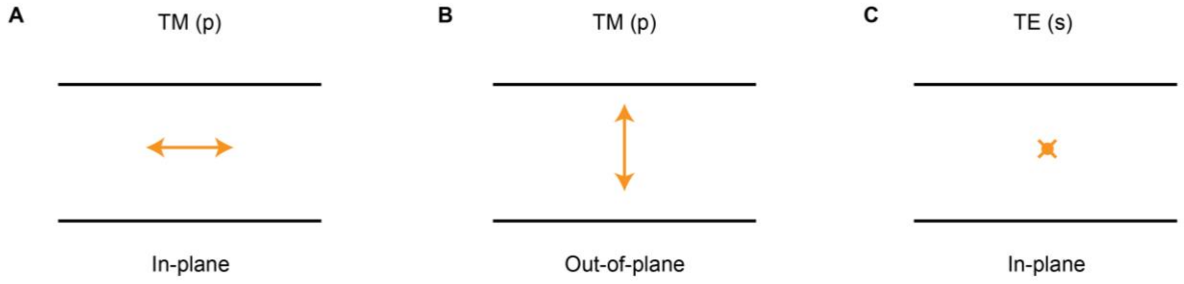

Fig. S2. Three different orientations of dipole in the two-dimensional plane.

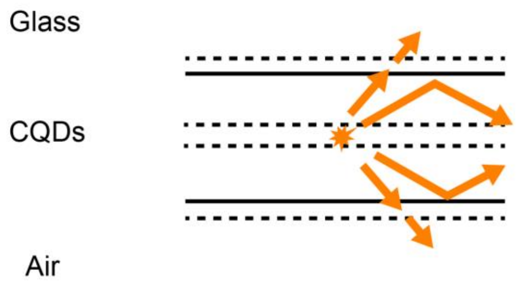

Fig. S3. The model used in BFP profile fitting.

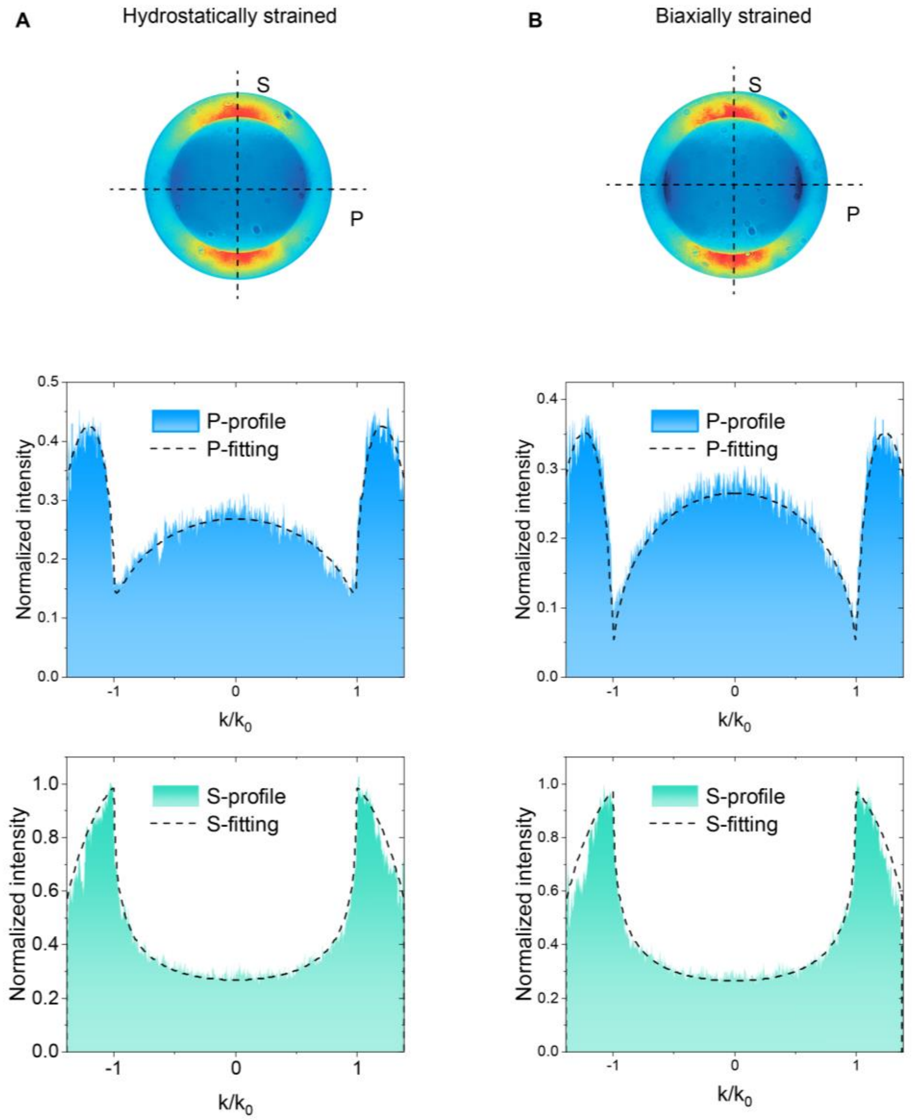

Fig. S4. The experimental back-focal plane images and corresponding p- and s- direction profiles. Figures correspond to (**A**) hydrostatically strained and (**B**) biaxially strained CQD films.

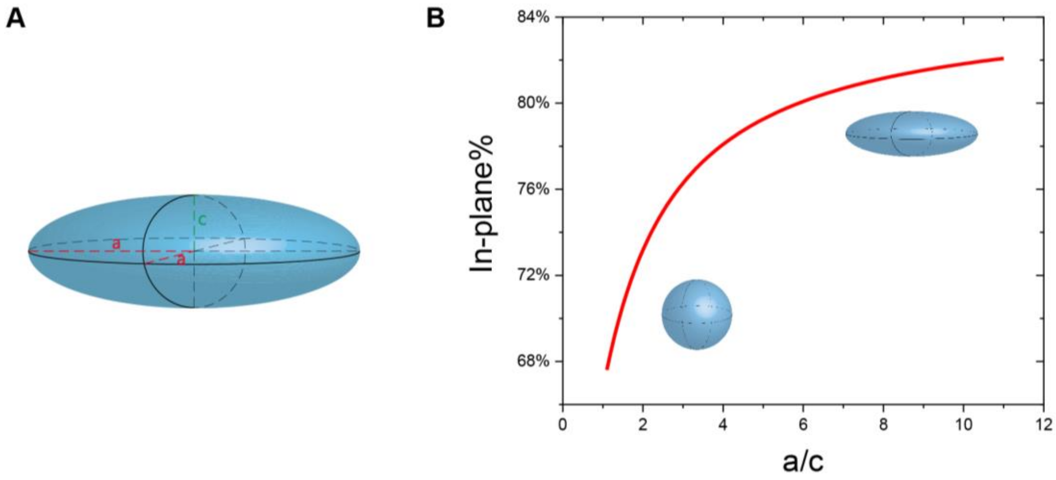

Fig. S5. The calculated dielectric effect for oblate ellipsoid. (**A**)The model for oblate ellipsoid. (**B**) According to the calculation, the pure dielectric effect is insufficient to yield 88% in-plane dipole proportion.

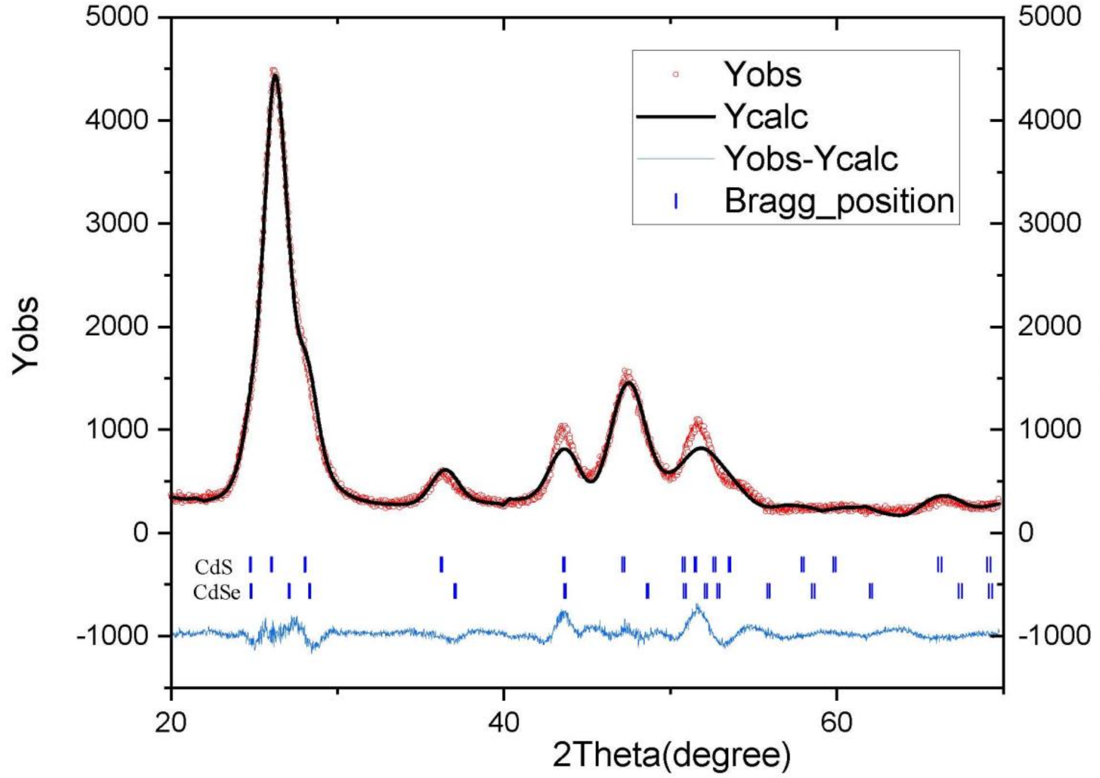

Fig. S6. The Rietvield refinement of XRD data. The preferred orientation parameters of the drop-coated film on the [002] crystal face can be obtained as: $G_1 = 0.7862$, $G_2 = 0.2105$. The $G_1 < 1$ represents that the sample is plate-shaped, and G_2 represents the proportion of the fraction that is not textured. It can be seen that the film has preferential orientation with [002] crystal facet perpendicular to the substrate.

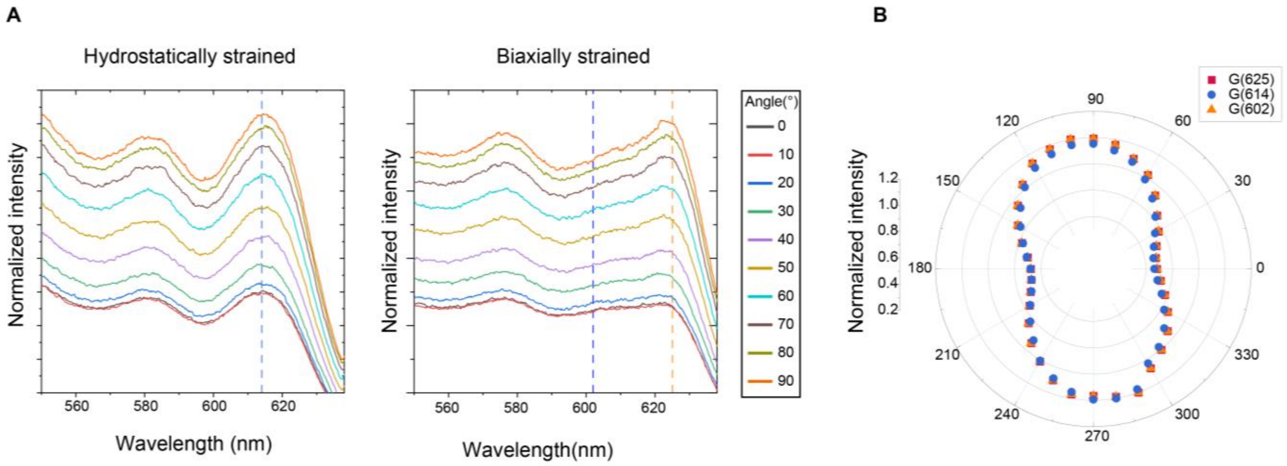

Fig. S7. The original data of polarization-dependent PLE spectra and the measured anisotropic factor. (**A**) PLE spectra of the hydrostatically strained and biaxially strained CQD films, which are corresponding to the original data of 0°-90° in fig. 3B. (**B**) The anisotropic factor G used to normalize the angle-dependent intensity in fig. 3B characterizes the intrinsic polarization anisotropy of the excitation monochromator.

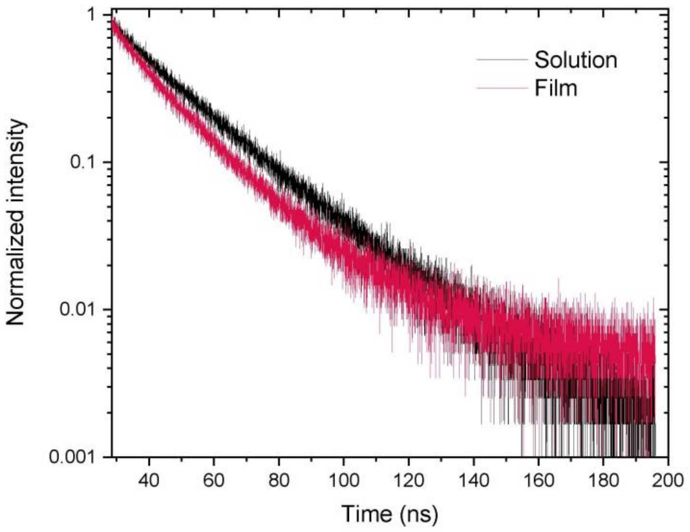

Fig. S8. The PL decay dynamics of CQDs film and CQDs dispersed in hexane. The similarity in lifetimes indicates that the CQDs film has comparable PLQY with the CQDs dispersed in solution, which is around 80%. The PL decay lifetime is measured by a TCSPC system consists of a 405nm 5Mhz pulsed laser, a single photon counting module (Excelitas SPCM-AQRH-W1), and a time-to-digital converter.

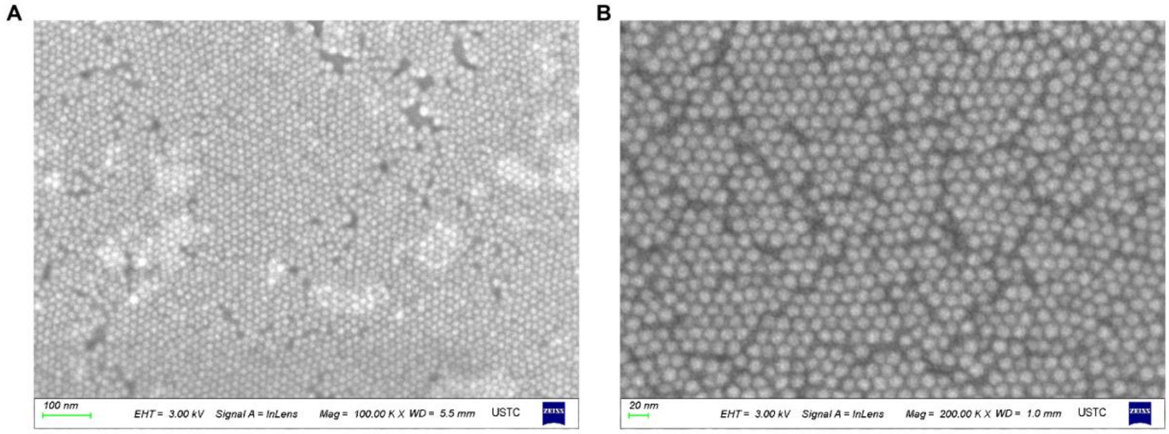

Fig. S9. The SEM images of sub-monolayer biaxially strained CQD film on glass substrate.

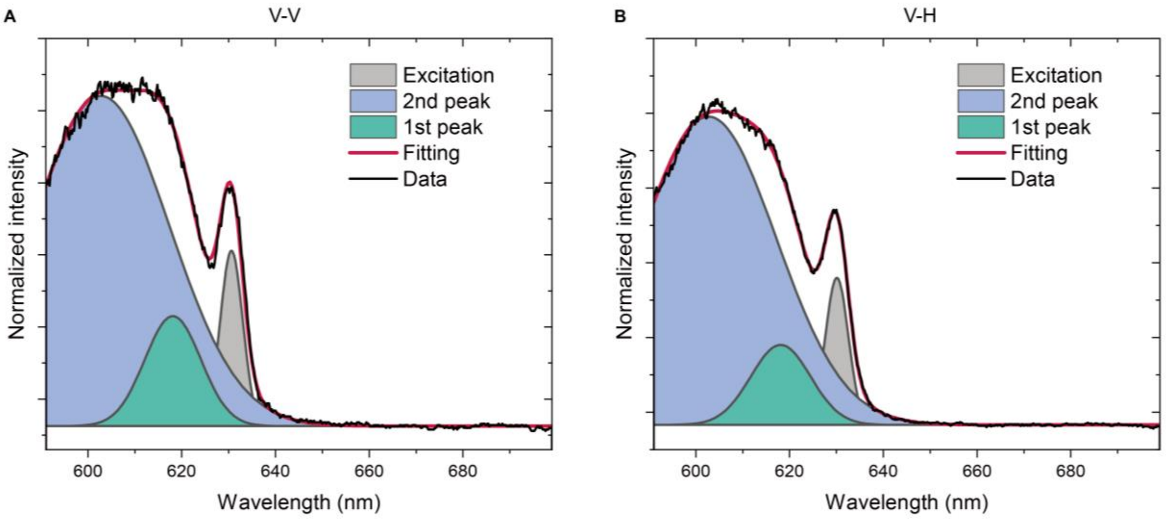

Fig. S10. The polarized absorption of biaxially strained CQDs dispersed solution. Figures shows the PLE spectra measured with the V-V (**A**) and V-H (**B**) polarized excitation-emission configuration (CQDs are dispersed in polybutadiene to slow down their rotation). The fitted area ratios of 1st to 2nd peak are 0.168 for V-V and 0.141 for V-H configurations, indicates the different polarization of 1st and 2nd absorption peaks. The PLE measurement is performed on the Hitachi F-7100 fluorescence spectrometer, and the polarization is introduced by two polarizers (Thorlabs WP25M-VIS).

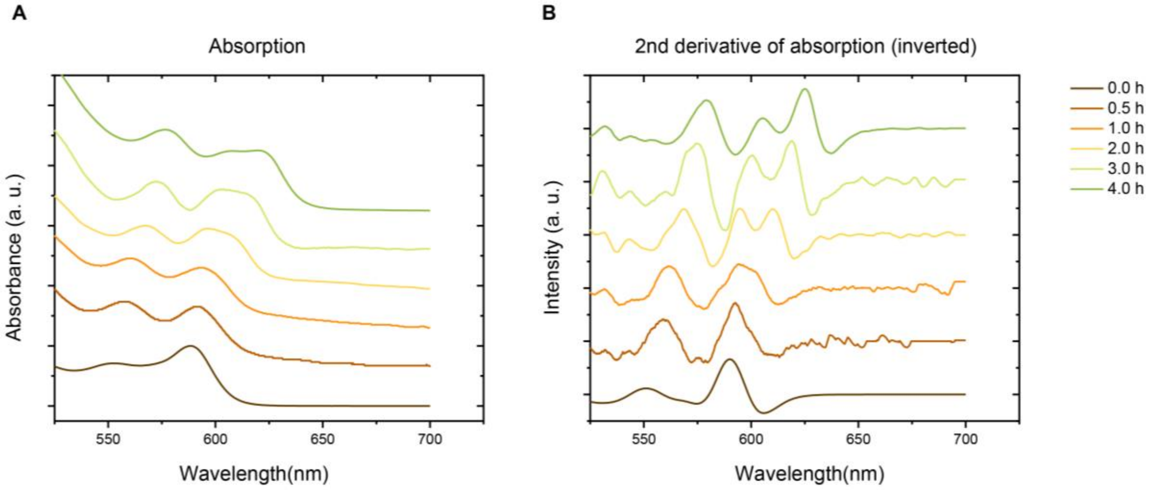

Fig. S11. The band-edge exciton peak splitting of biaxially stained CQDs. (**A**) The splitting increases with increasing the asymmetric shell thickness. (**B**) The second derivative of spectra in (A).

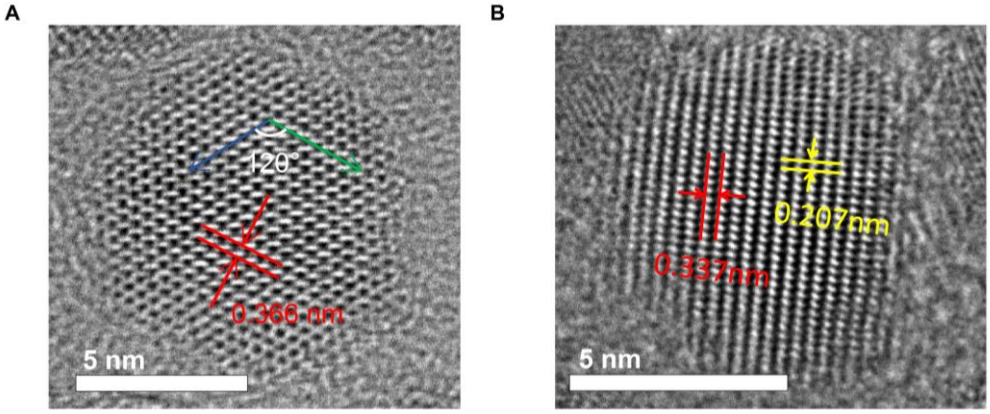

Fig. S12. High-resolution TEM of biaxially strained CQD. Observed along (**A**) [002] and (**B**) [1$\bar{1}$0] zone axes, the CQDs have similar size, confirming the near-spherical hat-like appearance.

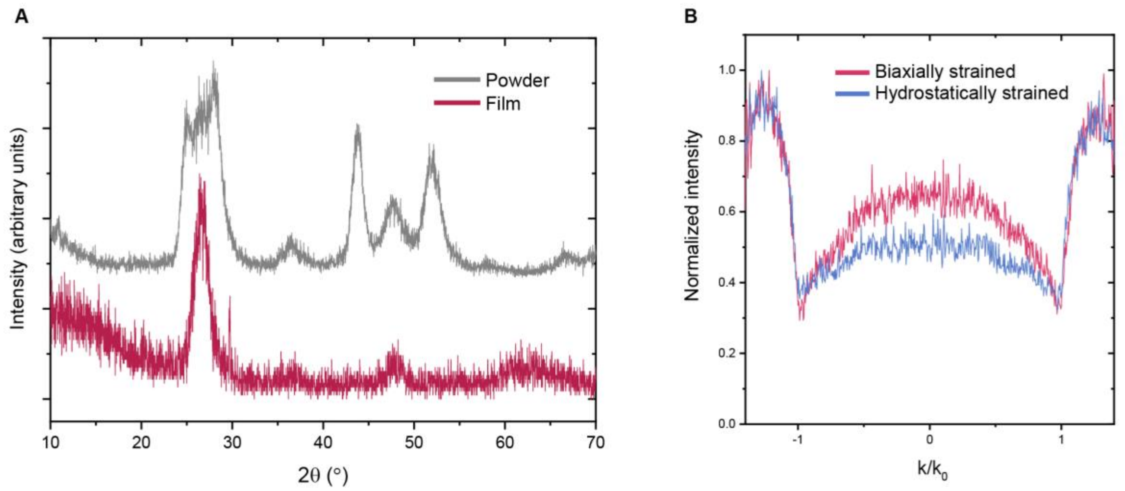

Fig. S13. The orientation characterization of biaxially strained CQD on TFB (poly[(9;9-dioctyluorenyl-2,7-diyl)-co-4,4-(N-(4-s-butylphenyl)diphenyl-amine)]) substrate. (A) XRD pattern of biaxially strained CQDs drop-casted on the TFB surface (non-diffraction silicon substrate), the powder XRD pattern is also provided for comparison. (B) the back-focal-plane (BFP) characterization of biaxially and hydrostatically strained CQD films on the TFB-glass substrates, respectively.

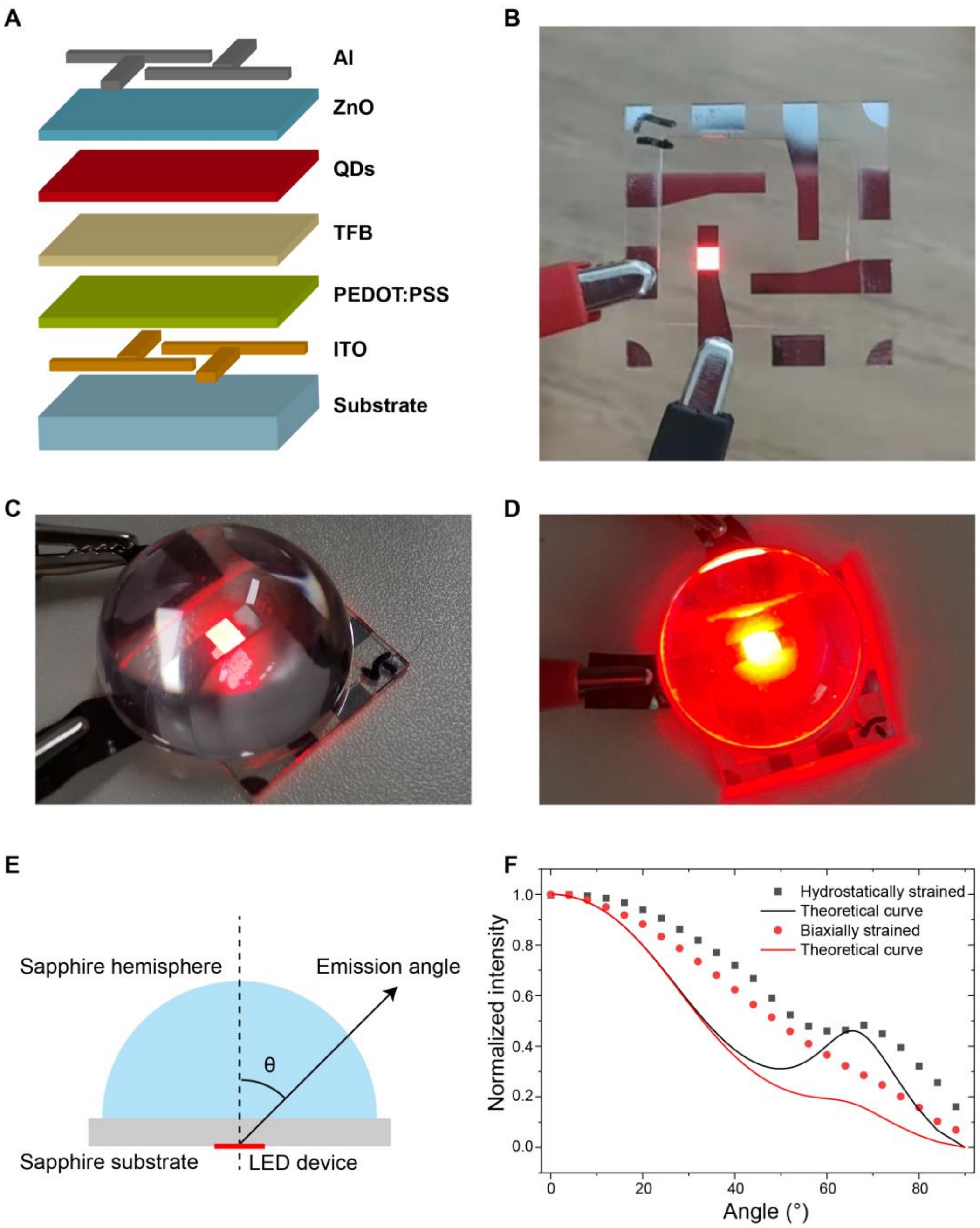

Fig. S14. The illustrations of the QD-LED devices and the electroluminescent angle-resolved intensity measurements. (A) The schematic LED structure used in the related electroluminescent experiments. (B) The picture of a lighted LED device. (C) and (D) is the picture of the device setup of the electroluminescent angle-resolved intensity measurements, which is composed of the sapphire hemispherical lens and the LED device. (E) The schema of electroluminescent angle-resolved intensity measurements: A sapphire hemisphere is connected to substrate through the index-matching oil (Cargile 1815Y, n=1.78), and the emission is collected from varying angles. (F) The experimental and theoretical emission patterns of devices for two kinds of QDs. The theoretical curves correspond to the 70% in-plane dipole proportion (black) and 88% in-plane dipole proportion (red), and the emission pattern simulation of LED device is the same as the method described in the supplementary text 1 and text 2.

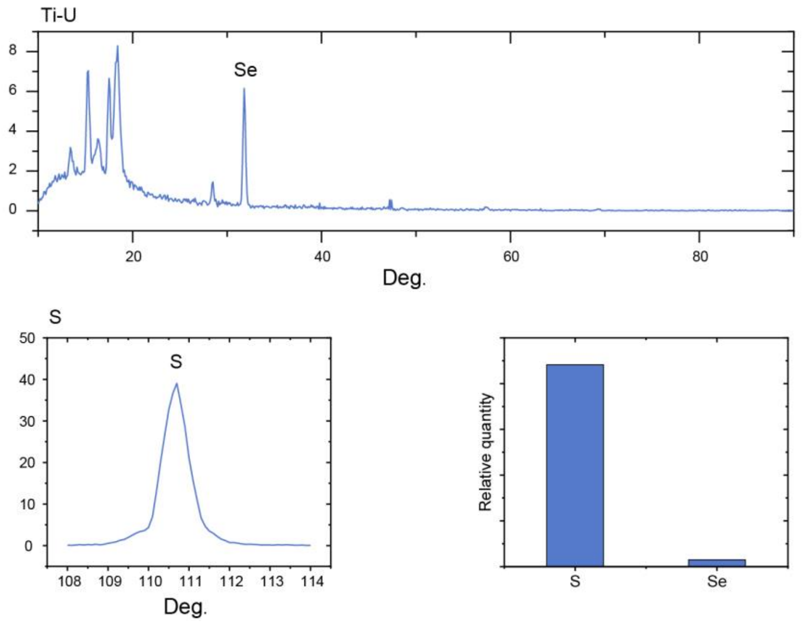

Fig. S15. The X-ray fluorescence (XRF) data of biaxially strained CQDs. The quantity ratio of S to Se is ~29.82, which indicates the size of CdSe core is about 1/3 of the whole quantum dot. This data is collected by a SHIMADZU XRF-1800 Spectrometer from the CQDs film on the silicon substrate.

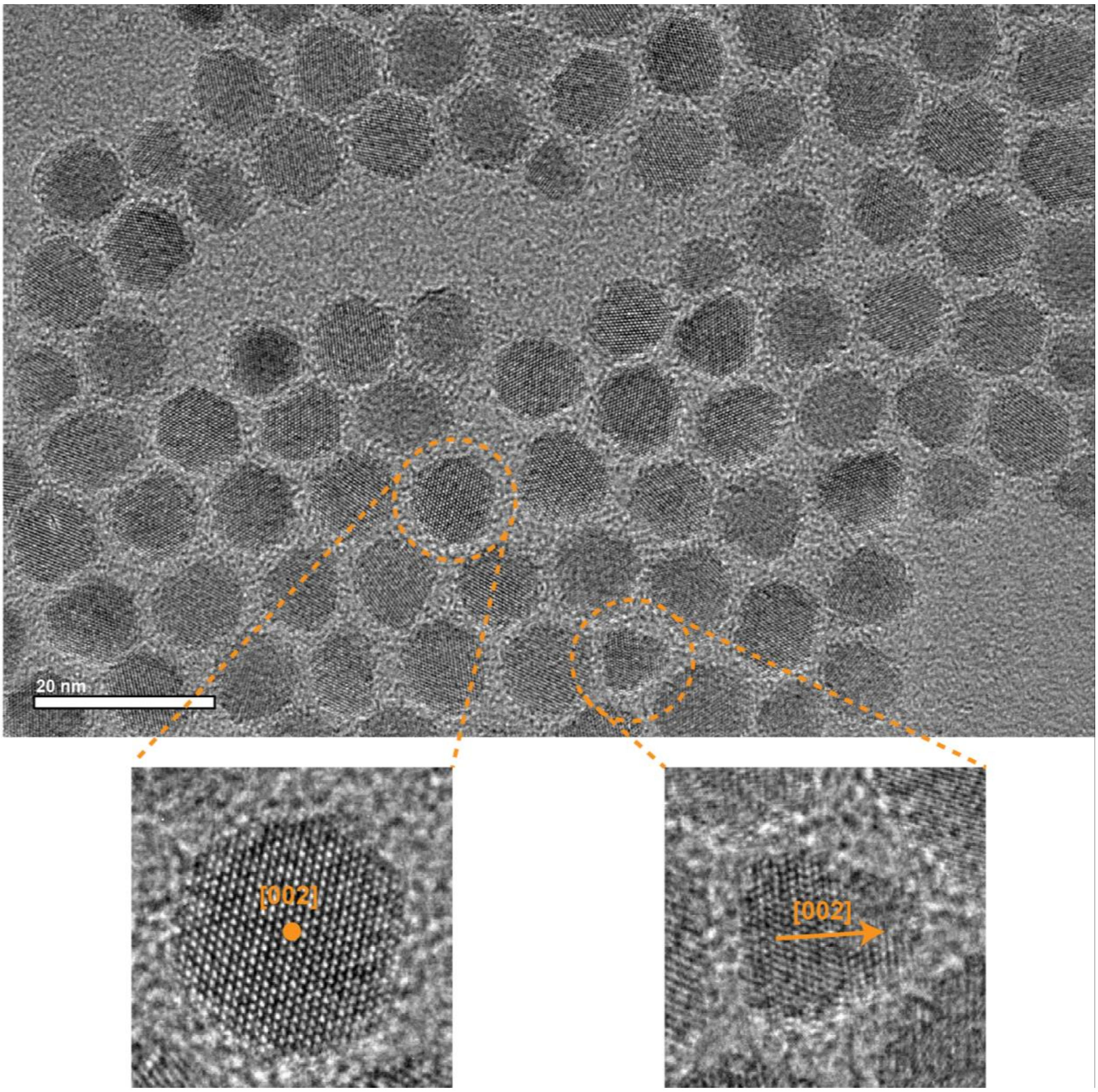

Fig. S16. The HRTEM image of biaxially strained CQDs. Almost all of quantum dots with size ranging from 5~10nm tend to lie on the grid with their [002] axis perpendicular to the substrate. In contrast, those smaller quantum dots with elongated shape along [002], i.e. expose less (002) facet, tend to lie on the grid with their [002] parallel to the substrate.

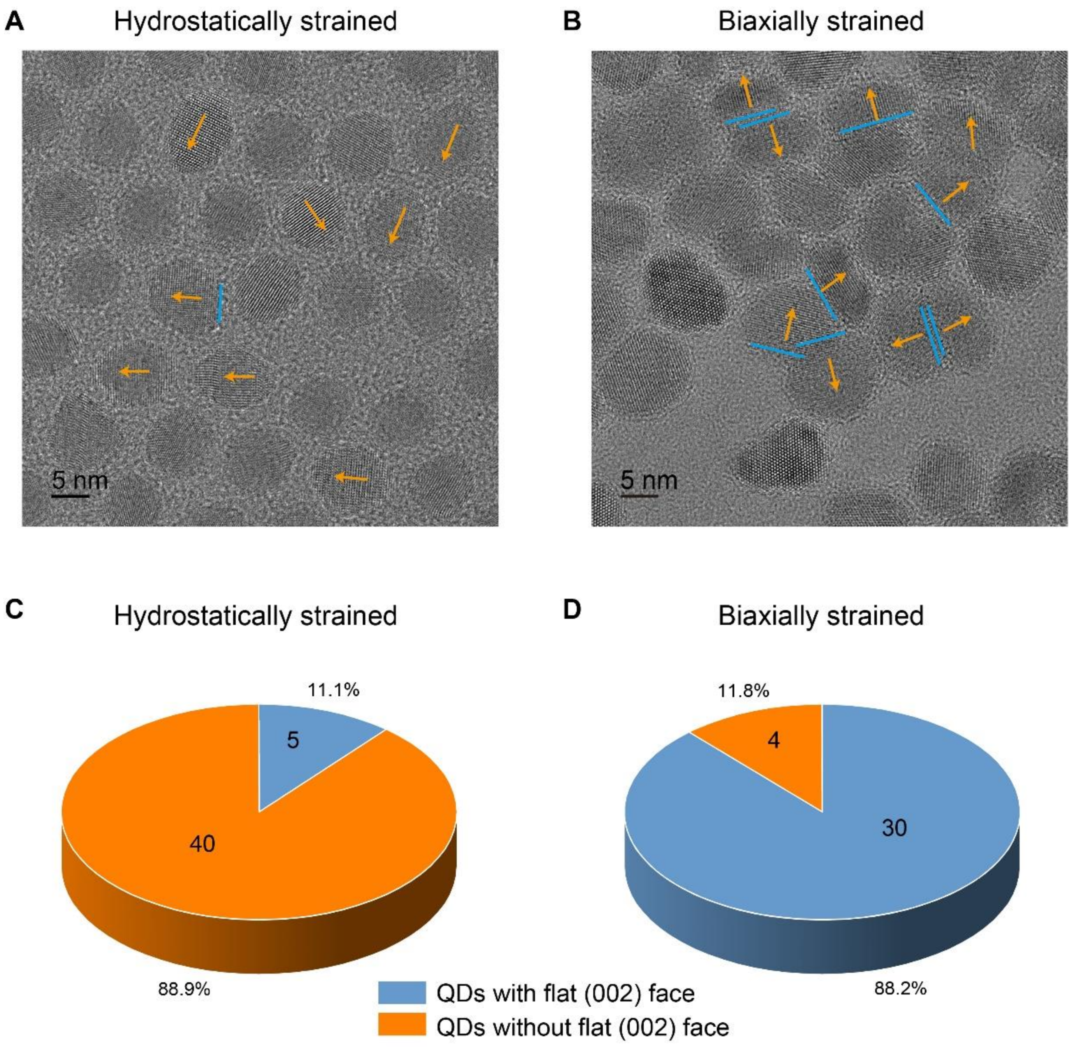

Fig. S17. The low magnification HRTEM images and the side-view shape statistics of the two kinds of QDs. The samples are prepared by spin-casting the QDs in chloroform dispersion to get the unassembled QDs film. (A) and (B) are the low magnification images showing the shape differences of hydrostatically and biaxially strained QDs, where the orange arrows represent the orientation of [002] axis (c-axis) determined by the lattice fringes and the blue lines represent the flat (002) face. (C) and (D) are the statistics for the shape of QDs among all the HRTEM images, and the counts are marked in the figures. The identifying criterion of flat (002) face is that the length of the face perpendicular to c-axis exceeds 5 nm.

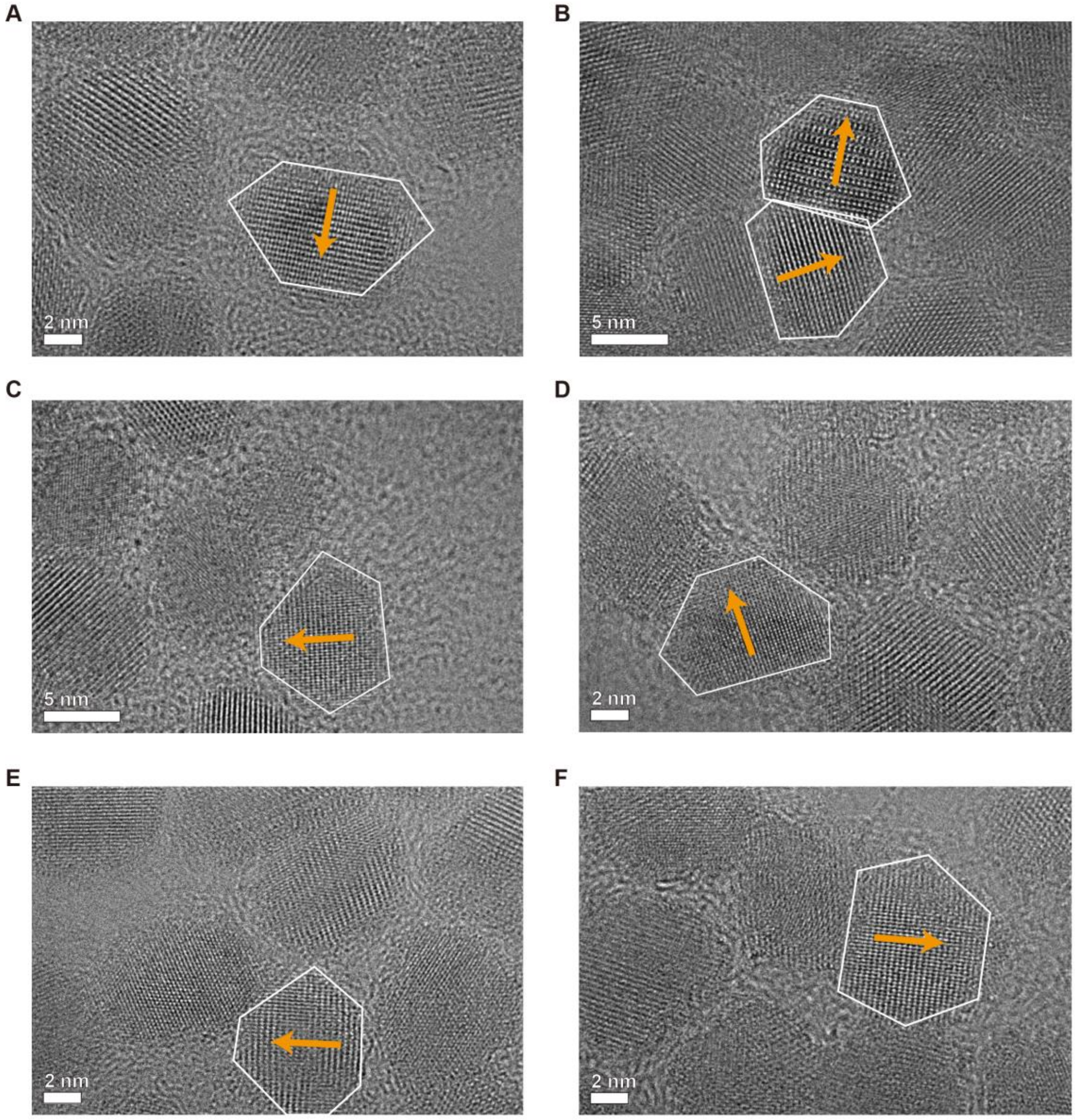

Fig. S18. The high magnification HRTEM images of the side-viewed biaxially strained QDs. The lattice fringes are analyzed and confirm the [002] axis orientation, and the shape of QDs are outlined with white lines.

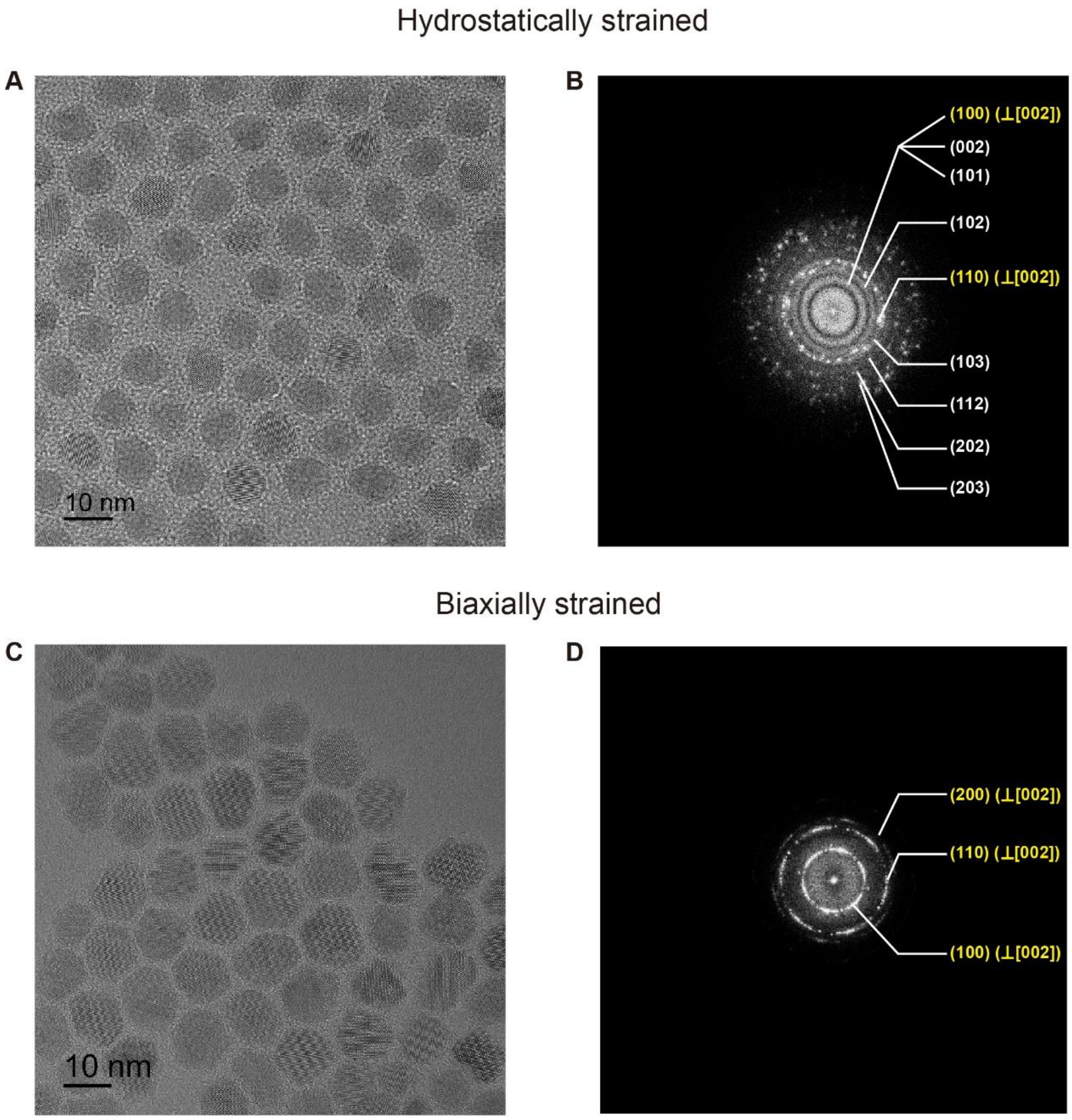

Fig. S19. The HRTEM and corresponding FFT of the spin-casted QDs film. (A) (B) and (C) (D) are the HRTEM and FFT images of hydrostatically strained QDs and biaxially strained QDs, respectively, where each Bragg diffraction ring is marked by the corresponding crystal facet. The random orientation of hydrostatically strained QDs led to the various FFT diffraction rings in (B), while biaxially strained QDs shows only three clear diffraction rings in (D) corresponding to the three facet perpendicular to [002] axis, which indicates the oriented alignment of [002] axis of biaxially strained QDs.
